# Comparative Evaluation of Real-Time Screening PCR Assays for *Giardia duodenalis* and of Assays Discriminating the Assemblages A and B

**DOI:** 10.3390/microorganisms10071310

**Published:** 2022-06-28

**Authors:** Felix Weinreich, Andreas Hahn, Kirsten Alexandra Eberhardt, Simone Kann, Torsten Feldt, Fred Stephen Sarfo, Veronica Di Cristanziano, Hagen Frickmann, Ulrike Loderstädt

**Affiliations:** 1 Department of Microbiology and Hospital Hygiene, Bundeswehr Hospital Hamburg, 20359 Hamburg, Germany; felixweinreich@bundeswehr.org (F.W.); frickmann@bnitm.de (H.F.); 2 Department of Medical Microbiology, Virology and Hygiene, University Medicine Rostock, 18057 Rostock, Germany; andreas.hahn@uni-rostock.de; 3 Department of Tropical Medicine, Bernhard Nocht Institute for Tropical Medicine & I. Department of Medicine, University Medical Center Hamburg-Eppendorf, 20251 Hamburg, Germany; k.eberhardt@bnitm.de; 4 Division of Hygiene and Infectious Diseases, Institute of Hygiene and Environment, 20539 Hamburg, Germany; 5 Medmissio, 97074 Würzburg, Germany; simone.kann@medmissio.de; 6 Department of Gastroenterology, Hepatology and Infectious Diseases, University Medical Center Düsseldorf, 40225 Düsseldorf, Germany; torsten.feldt@med.uni-duesseldorf.de; 7 Department of Medicine, Kwame Nkrumah University of Science and Technology, Kumasi AK-4944, Ghana; stephensarfo78@gmail.com; 8 Institute of Virology, Faculty of Medicine and University Hospital of Cologne, University of Cologne, 50935 Cologne, Germany; veronica.di-cristanziano@uk-koeln.de; 9 Department of Hospital Hygiene & Infectious Diseases, University Medicine Göttingen, 37075 Göttingen, Germany

**Keywords:** giardiasis, test comparison, molecular diagnosis, sensitivity, specificity, latent class analysis

## Abstract

Due to superior sensitivity compared to traditional microscopy, real-time PCR has been well established for the diagnosis of *Giardia duodenalis* in human stool samples. In this study, screening real-time PCRs for different target genes of *G. duodenalis*, i.e., the 18S rRNA gene, the *gdh* (glutamate dehydrogenase) gene and the *bg* (beta-giardin) gene, were comparatively assessed next to various real-time PCR assays for the discrimination of the assemblages A and B of *G. duodenalis* targeting the *bg* gene with and without locked nucleic acid–containing probes as well as the *tpi* (triose phosphate isomerase) gene. The screening PCRs were assessed by including 872 non-preselected samples with a high pre-test probability for *G. duodenalis* in the statistical analysis, while 53 *G. duodenalis*-positive samples as indicated by at least two screening PCRs were finally included in the assessment of the assemblage-specific PCRs. For the screening PCRs, sensitivity estimated with latent class analysis (LCA) ranged from 17.5% to 100%, specificity from 92.3% to 100% with an accuracy-adjusted prevalence of 7.2% for *G. duodenalis* within the non-preselected sample collection. In detail, sensitivity and specificity were 100% and 100% for the 18S rRNA gene-specific assay, 17.5% and 92.3% for the *gdh* gene-specific assay, and 31.7% and 100% for the *bg* gene-specific assay, respectively. Agreement kappa was slight with only 15.5%. For the assemblage-specific PCRs, estimated sensitivity ranged from 82.1% to 100%, specificity from 84.0% to 100% with nearly perfect agreement kappa of 90.1% for assemblage A and yet substantial agreement of 74.8% for assemblage B. In detail for assemblage A, sensitivity and specificity were 100% and 100% for the *bg* gene-specific assay without locked nucleic acids (LNA) as well as 100% and 97.8% for both the *bg* gene-specific assay with LNA and the *tri* gene-specific assay, respectively. For assemblage B, sensitivity and specificity were 100% and 100% for the *bg* gene-specific assay without LNA, 96.4% and 84.0% for the *bg* gene-specific assay with LNA, and 82.1% and 100% for the *tri* gene-specific assay, respectively. Within the assessed sample collection, the observed proportion comprised 15.1% *G. duodenalis* assemblage A, 52.8% *G. duodenalis* assemblage B and 32.1% non-resolved assemblages. Only little differences were observed regarding the cycle threshold (Ct) values when comparing the assays. In conclusion, best diagnostic accuracy was shown for an 18S rRNA gene-specific screening assay for *G. duodenalis* and for a differentiation assay discriminating the *G. duodenalis* assemblages A and B by targeting the *bg* gene with probes not containing locked nucleic acids. By adding additional highly specific competitor assays for confirmation testing, diagnostic specificity can be further increased on the cost of sensitivity if optimized specificity is desired.

## 1. Introduction

*Giardia duodenalis* is an enteric protozoan parasite causing human disease with a symptom spectrum ranging from acute diarrhea and abdominal pain to chronic malabsorption and weight loss [1,2]. Transmission occurs via the fecal–oral route [3] with increased proportions of cases in returnees from resource-limited regions where adequate hygiene precautions cannot be maintained for economic reasons [2,4,5,6,7,8,9]. In such resource-poor endemic regions, asymptomatic courses are frequently observed [10,11]. While nitroimidazoles are still used as the therapy of first choice, considerable resistance rates complicate the antiparasitic therapy and can be even aggravated in case of insufficient compliance of the patients with the prescribed medication [2].

While microscopy was the traditional diagnostic reference standard, it has been repeatedly confirmed that higher sensitivity and lower investigator-dependence of real-time PCR can be considered well-established [12,13] at least for resource-rich non-endemicity settings. Under such circumstances, sensitivity and specificity of microscopy for the diagnosis of *G. duodenalis* in human stool samples were recently estimated as 72% and 99%, respectively [13], while sensitivity and specificity of various compared investigator-designed and commercial real-time PCR assays ranged from 90% to >99% and from 76% to virtually 100%, respectively [13,14]. Of note, however, almost perfect agreement (Fleiss kappa of 0.861) of positive results was recorded in a study comparing microscopy and various real-time PCRs [13] next to substantial agreement (Fleiss kappa of 0.653) of positive results in a head-to-head comparison just between different real-time PCR assays [14] according to the criteria by Landis and Koch [15]. The observed discordance between the PCR assays could be due to various reasons, including the choice of the target sequences and the choice of the oligonucleotides for the amplification [16,17] next to other conditions such as sample storage and transport [18] or the mode of nucleic acid extraction [19].

Typing approaches below the species level have shown that the species *G. duodenalis* comprises the assemblages A–H [20,21,22], of which the assemblages A and B are considered as zoonotic and of relevance for human disease [20,23]. Thereby, the identification of virulence factors for the discrimination of mere colonization and true infection is still an issue of ongoing research [23].

Various DNA sequence targets have been tested and applied for real-time PCR-based screening for *G. duodenalis* as well as for diagnostic discrimination of the etiologically relevant assemblages A and B in both validation studies and epidemiological assessments, including genes encoding beta-giardin (*bg*), triose phosphate isomerase (*tpi*), 18S rRNA (=the small sub-unit of ribosomal RNA), and glutamate dehydrogenase (*gdh*) [19,20,24,25,26,27,28,29,30,31,32,33,34,35,36]. In this study, three previously described real-time PCR screening assays for *G. duodenalis* targeting the 18S rRNA gene, the *bg* gene and the *gdh* gene, respectively [25,26,27], as well as three described real-time PCR differentiation assays targeting the *bg* gene (*n* = 2 assays) and the *tpi* gene [25,28,29] were compared in head-to-head test comparisons without a reference standard applying latent class analysis [16,37]. Our overarching objective was to assess the influence of the choice of different target genes on the diagnostic accuracy of real-time PCR for *G. duodenalis* and its assemblages A and B.

## 2. Materials and Methods

### 2.1. Residual Sample Materials Used for the Test Comparison, Inclusion and Exclusion Criteria

The study was conducted as a comparative head-to-head assessment of different real-time-PCR assays with historical residual sample materials without microscopic characterization. For the comparison of the screening PCR assays targeting *G. dudodenalis*, residual volumes of nucleic acid extractions from stool samples of 905 Ghanaian HIV patients from previous epidemiological and technical assessments [38,39,40,41,42,43] were used, so an acceptable pre-test probability due to a high prevalence of giardiasis in Ghana could be assumed [44,45]. All available residual sample materials with sufficient volumes were included. After testing, samples showing PCR inhibition in the inhibition control PCR as detailed below were excluded from the assessments. For the subsequent comparison of the assemblage-specific duplex real-time PCR assays, 55 residual volumes of nucleic acid extractions from samples with positive screening PCR results for *G. duodenalis* from previous epidemiological studies [38,39,40,41,42,43,46] were applied. Inclusion criterion was that at least two out of three *G. duodenalis*-specific screening PCR assays provided a positive signal, making abundance of *G. duodenalis* DNA likely. Again, samples showing PCR inhibition in the inhibition control PCR as detailed below were excluded from the assessments after testing. In line with the ethical clearance demanding an anonymized use of residual sample materials for test comparison purposes, no patient-specific data can be provided, which is an admitted deviation from the STARD (Standards for Reporting of Diagnostic Accuracy) criteria [47].

### 2.2. Nucleic Acid Extraction and Storage

Nucleic acid extraction of the included stool samples was performed applying the QIAamp stool DNA mini kit (Qiagen, Hilden, Germany) as described by the manufacturer and others [48]. Prior to the PCR assessments, the nucleic acid extractions were stored between a few months up to 15 years and deep-frozen at −80 °C in order to preserve the nucleic acid quality within the samples.

### 2.3. Real-Time Screening PCR Assays for Giardia duodenalis and Differentiation Assays for the Assemblages A and B

The assessed real-time screening PCRs for *G. duodenalis* targeted the 18S rRNA gene [26], the *gdh* (glutamate dehydrogenase) gene [27], and the *bg* (beta-giardin) gene [25], respectively. The compared duplex real-time PCRs for the discrimination of the *G. duodenalis* assemblages A and B targeted the *bg* gene excluding [25] and including [28] the use of LNA (locked nucleic acid)–containing probes as well as the *tpi* (triose phosphate isomerase) gene [29], respectively. Real-time PCR for Phocid herpes virus (PhHV) DNA was applied as an internal control as described [48]. The applied oligonucleotides are shown in the Appendix A Table A1. The assays were in parallel established on RotorGene Q cyclers (Qiagen, Hilden, Germany) and on the technically similar magnetic induction cycler (MIC, Bio Molecular Systems Ltd., London, UK). On both devices, they showed comparable characteristics. The protocols were run as described [25,26,27,28,29] with minor modifications as indicated in the Appendix A Table A2. Plasmid-based positive controls (plasmid insert sequence shown in the Appendix A Table A3) and PCR-grade water-based negative controls were included in each run. The cycle threshold (Ct) values of the positive controls were expected in a range of ±2 Ct steps. Each assessed residual sample material was run once in each assay, thus simulating diagnostic real-life conditions. With a dilution series of the positive control PCR plasmid, detection thresholds of the assessed real-time PCRs were recorded as follows: A limit of detection of less than 10 copies per µL eluate was recorded for the real-time PCR screening assays targeting the 18S rRNA gene, the *bg* gene and the *gdh* gene as well as for the real-time PCR differentiation assays targeting the *tpi* gene and the *bg* gene. For the PCR differentiation assay targeting the *bg* gene with locked nucleic acid probes, a slightly higher limit of detection of 83 copies per µL was observed.

### 2.4. Diagnostics Accuracy Estimation, Agreement, and Comparison of Obtained Cycle Threshold (Ct) Values

Latent class analysis [16,37], which is a variant of structural equation models which aims at estimating latent non-observed variables as the actual disease status over observed variables, such as the results of diagnostic test assays, was applied for the estimation of the diagnostic accuracy parameters sensitivity and specificity of all assessed assays without a reference standard. Further, diagnostic accuracy–adjusted prevalence estimation was conducted using this approach. Agreement according to Fleiss’ kappa of positive results obtained with the compared assays was calculated and interpretated as reported elsewhere [15]. Recorded cycle threshold (Ct) values were descriptively compared. The software Stata/IC 15.1 for Mac 64-bit Intel (College Station, TX, USA) was used for the calculations.

### 2.5. Ethics

Ethical clearance allowing the use of anonymized residual sample materials for test comparison purposes without requirement of informed consent was obtained from the medical association of Hamburg, Germany (reference number: WF-011/19, provided on 11 March 2019), according to national German laws. The study was performed in line with the Declaration of Helsinki and its amendments.

## 3. Results

### 3.1. Sensitivity and Specificity of the Screening and Differentiation PCRs, Agreement between the Compared Assays, and Accuracy-Adjusted Prevalence Estimations

From the 905 samples without microscopic characterization assessed for the comparison of the *G. duodenalis*-specific screening PCRs, a total of 872 could be included in the calculations after exclusion of inhibited samples. When comparing the three assessed screening PCRs for *G. duodenalis*, only slight agreement was recorded (Table 1). As estimated applying latent class analysis (LCA), best sensitivity was calculated for the 18S rRNA gene PCR followed by the *bg* gene-specific PCR and the *gdh* gene-specific PCR in declining order. The lower sensitivity of the *gdh* gene-specific PCR compared to the *bg* gene-specific PCR in spite of more positive signals in the *gdh* gene-specific PCR results from this assay’s lower specificity as calculated applying LCA. Thereby, acceptable sensitivity in the >95% range could be recorded for the 18S rRNA gene PCR alone. Regarding specificity, near-perfect specificity was estimated for the 18S rRNA gene PCR and the *bg* gene-specific PCR, while with >95% margin was slightly missed by the *gdh* gene-specific PCR. A cross-table indicating the matches and mismatches regarding the positive results is shown in the Appendix A Table A4. Diagnostic accuracy–adjusted prevalence estimation indicated a *G. duodenalis* prevalence of 7.2% within the assessed study population.

After applying the inclusion criteria of at least two positive *G. duodenalis*-specific PCRs and after exclusion of the inhibited samples, a total of 53 samples could be included in the comparisons of the assemblage-specific PCR assays (Table 2). For the three assays targeting assemblage A, nearly perfect agreement could be demonstrated with an estimated sensitivity of 100%. Regarding specificity, 100% specificity was calculated for the *bg* gene-specific assay without LNA, while a slightly reduced specificity still over the >95% margin was calculated for the *bg* gene-specific assay with LNA and for the *tri* gene-specific assay. A proportion of 15.1% assemblage A was calculated applying diagnostic accuracy–adjusted prevalence estimation. A cross-table indicating the matches and mismatches of positive PCR results for *G. duodenalis* assemblage A is shown as Appendix A Table A5.

Focusing on assemblage B, agreement between the compared PCR assays was yet substantial (Table 2). As estimated by LCA, sensitivity declined in the order of the *bg* gene-specific assay without LNA, the *bg* gene-specific assay with LNA and the *tri* gene-specific assay. The *bg* gene-specific assay both with and without LNA were still above the >95% sensitivity margin, while this was not the case for the *tri* gene-specific assay. While specificity of 100% was calculated for *bg* gene-specific assay without LNA and the *tri* gene-specific assay, a specificity considerably below the >95% margin was estimated for the *bg* gene-specific assay with LNA. Diagnostic accuracy–adjusted prevalence estimation allowed the calculation of 52.8% assemblage B among the 53 included samples. In the Appendix A Table A6, a cross-table indicating matching and mismatching positive PCR results for the assemblage B is shown.

### 3.2. Comparison of the Recorded Cycle Threshold Values with the Assessed Screening and Differentiation PCRs

Regarding the positive results in the screening PCR assays, comparable mean Ct values were recorded for the 18S rRNA gene PCR and the *bg* gene PCR, while a tendency for higher Ct values was seen for the *gdh* gene PCR. If not the mean but the median values were assessed, lowest Ct value were seen for the *bg* gene PCR while the median Ct values of the 18S rRNA gene PCR and the *ghd* gene PCR were within a similar range (Table 3).

Focusing on the assemblage specific PCRs, a weak tendency for lower Ct values was seen for the *bg* gene-specific assay without LNA only with quite similar Ct values for the *bg* gene-specific assay with LNA and the *tri* gene-specific assay. This applied both for the mean and the median values (Table 4).

## 4. Discussion

The study consisted of two parts. In the first part, screening PCRs for *G. duodenalis* with different target genes [25,26,27] were assessed in non-preselected samples with a high pre-test probability of positivity which was reflected by a diagnostic-accuracy adjusted *G. duodenalis* prevalence of 7.2%. This part of the study was primarily performed to identify samples with a high likelihood of being positive for *G. duodenalis*, because microscopic characterization was missing. Indeed, considerable discrepancy of the diagnostic accuracy of the three compared screening assays confirmed discordance as recorded in previous comparison studies [13,14]. Not surprisingly and reflecting the previous results [13,14], best sensitivity was estimated for the 18S rRNA gene as a PCR target occurring in multiple copies in the genome of *G. duodenalis*. The poorer sensitivity of the *gdh* gene-specific assay but not of the *bg* gene-specific assay was associated with a tendency for higher Ct values. Interestingly, the *gdh*-gene specific assay also scored worse regarding specificity compared to the 18S rRNA gene-specific assay and the *bg* gene-specific assay, also confirming previous results which suggest the ribosomal sub-unit gene-specific assay to be best suited for *G. duodenalis*-specific screening approaches [13,14]. Accordingly, one might speculate that the higher Ct values observed with the *gdh* gene-specific assay could be associated with false positive reactions in line with this assay’s comparably low specificity suggested by latent class analysis. This also explains the *gdh* gene-specific assay’s particularly poor sensitivity in spite of a comparably high number of 73 positive signals.

In the second part of the study, samples showing positive results in at least two screening PCRs, so true positivity for *G. duodenalis* was considered as highly likely, were subjected to various differentiation PCRs targeting the *G. duodenalis* assemblages A and B. Again, three different assays per assemblage were compared [25,28,29]. However, two assays targeted the same gene but were different by the use or non-use of LNA in order to affect the binding characteristics of the hybridization probes of the real-time PCR assays [25,28].

Focusing on those assemblage-specific assays, best results regarding sensitivity, specificity, and a tendency for lower Ct values were estimated for the *bg* gene-specific assay without LNA for both assemblages [25]. While both the *bg* gene-specific assay with LNA [28] and the *tri* gene-specific assay [29] still showed acceptable accuracy for the assemblage A, this situation was different for the assemblage B. The *bg* gene-specific assay with LNA targeting the assemblage B showed insufficient specificity, and insufficient sensitivity was observed for the respective *tri* gene-specific assay. Interestingly, however, the *bg* gene-specific assay with LNA did not show relevantly decreased sensitivity, although the calculated copy numbers defining its limit of detection were slightly higher than those calculated for the other assays.

The study has a number of limitations. First and most importantly, the study was performed with residual sample materials and did not include microscopic assessments. To reduce the risk of relevant bias due to potential specificity issues of the screening PCRs at least for the assessment of the assemblage-specific assays, only samples with positive results in at least two different screening assays were included in those assessments. Second and resulting from this strategy, only a limited number of samples could be included in the assessment of the assembly-specific assays. In particular for assemblage A, only single-digit numbers of positive results were shown by the different assays. With an estimated proportion of 15.1% of samples being positive for assemblage A and 52.8% being positive for assemblage B, adjustment of an assemblage failed in 32.1% of the instances. Hence, it remains uncertain whether this lack of adjustment resulted from a lack of sensitivity of the assays or from the fact that respective *G. duodenalis* strains detected by the screening PCRs were from assemblages other than A or B. Third, due to the fact that frozen residual samples materials were used for the study, it cannot be excluded that DNA degradation occurred in spite of appropriate storage at −80 °C. However, all assessments of this study were performed in temporal proximity with each other, and so it can be assumed that the conditions were virtually the same for all competing assays. Fourth, the assessed primer–probe combinations just represented an exemplarily chosen sub-set of available real-time PCR protocols, while more respective assays have recently been introduced [49]. Restricted volumes of residual sample materials made this choice necessary. Accordingly, no conclusions on how the assessed assays would have scored in direct comparison to other described ones [49] can be drawn based on the presented data.

## 5. Conclusions

In spite of the above-mentioned limitations of its interpretation, the study suggests that the *G. duodenalis* 18S rRNA gene-based screening assay and the assemblage-specific *bg* gene assay without LNA are associated with high diagnostic accuracy. If increased diagnostic specificity is desired and associated lower sensitivity is thereby accepted, confirmation testing with highly specific assays such as the *bg* gene-specific screening assay, any other of the assessed assemblage A-specific assays, and the *tri* gene-specific assemblage B assay can be considered.

## Figures and Tables

**Table 1:** Agreement kappa between the compared real-time screening PCR assays targeting *G. duodenalis* as well as sensitivity, specificity, and accuracy-adjusted prevalence as calculated with latent class analysis (LCA) based on the assessment of 872 non-inhibited samples with high pre-test probability.

Assay	Positives (%)	Sensitivity (0.95 CI)	Specificity (0.95 CI)	Kappa (0.95 CI)
18S rRNA gene	63 (7.22)	1 (0, 1)	1 (n.e.)	0.155 (0.110, 0.205)
*gdh*	73 (8.37)	0.175 (0.099, 0.288)	0.923 (0.903, 0.940)	
*bg*	20 (2.29)	0.317 (0.215, 0.441)	1 (n.e.)	
Prevalence (0.95 CI)	7.22% (5.69%, 9.14%)			

*n* = number included after exclusion of inhibited samples. n.e. = not estimable.

**Table 2:** Agreement kappa between the compared real-time differentiation PCR assays targeting the *G. duodenalis* assemblages A and B as well as sensitivity, specificity, and accuracy-adjusted prevalence as calculated with latent class analysis (LCA) based on the assessment of *n* = 53 non-inhibited samples testing positive in at least two of the different *G. duodenalis* screening PCRs.

Assay	Positives (%)	Sensitivity (0.95 CI)	Specificity (0.95 CI)	Kappa (0.95 CI)
*bg* of assemblage A without LNA	8 (15.09)	1 (0, 1)	1 (0, 1)	0.908 (0.737, 1)
*bg* of assemblage A with LNA	9 (16.98)	1 (0, 1)	0.978 (0.858, 0.997)	
*tri* of assemblage A	9 (16.98)	1 (0, 1)	0.978 (0.858, 0.997)	
Prevalence (0.95 CI)	15.07% (7.72%, 27.36))			
*bg* of assemblage B without LNA	28 (52.83)	1 (0, 1)	1 (0, 1)	0.748 (0.622, 0.874)
*bg* of assemblage B with LNA	31 (58.49)	0.964 (0.786, 0.995)	0.840 (0.643, 0.940)	
*tri* of assemblage B	23 (43.40)	0.821 (0.636, 0.924)	1 (n.e.)	
Prevalence (0.95 CI)	52.82% (39.51%, 65.76%)			

**Table 3:** Recorded cycle threshold (Ct) values of the real-time screening PCR assays targeting *G. duodenalis*.

Assay	*n*	Mean (SD)	Median (IQR)
18S rRNA gene	63	28.74 (4.92)	29.91 (26.53, 32.53)
*gdh*	73	30.39 (2.20)	30.45 (28.84, 31.58)
*bg*	20	28.32 (3.45)	28.24 (26.58, 30.98)

*n* = number of samples. SD = standard deviation. IQR = interquartile range.

**Table 4:** Recorded cycle threshold (Ct) values of the assemblage-specific PCR assays.

Assay	*n*	Mean (SD)	Median (IQR)
*bg* of assemblage A without LNA	8	27.38 (3.50)	27.47 (23.95, 31.02)
*bg* of assemblage A with LNA	9	28.39 (4.13)	28.34 (26.60, 30.24)
*tri* of assemblage A	9	28.66 (3.93)	29.68 (24.52, 31.60)
*bg* of assemblage B without LNA	28	29.86 (3.45)	30.38 (28.14, 31.67)
*bg* of assemblage B with LNA	31	30.81 (3.83)	30.85 (28.16, 34.49)
*tri* of assemblage B	23	30.24 (3.16)	30.80 (28.38, 32.30)

*n* = number of samples. SD = standard deviation. IQR = interquartile range.

## Data Availability

All relevant data are presented in the article. Raw data can be provided on reasonable request.

## Appendix A

**Table A1:** Oligonucleotides applied for the compared real-time PCR assays.

Target Pathogen	Target Gene	Forward Primer Sequence	Reverse Primer Sequence	Probe Sequence	Reference, Where the Detailed Protocol Can Be Found
*Giardia duodenalis*	*bp*	5′-CATCCGCGAGGAGGTCAA-3′	5′-GCAGCCATGGTGTCGATCT-3′	5′-AAGTCCGCCGACAACATGTACCTAACGA-3′	[25]
*Giardia duodenalis*	18S rRNA	5′-GACGGCTCAGGACAACGGTT-3′	5′-TTGCCAGCGGTGTCCG-3′	5′-CCCGCGGCGGTCCCTGCTAG-3′	[26]
*Giardia duodenalis*	*gdh*	5′-CTGAAGAACTCCCTCACCAC-3′	5′-CAGAAGCGCATGACCTCGTTG-3′	5′-CAAGGGCGGCTCCGACTTTGACCCAA-3′	[27]
*Giardia duodenalis* assemblage A	*bp*	5′-CCTCAAGAGCCTGAACGATCTC-3′	5′-AGCTGGTCGTACATCTTCTTCCTT-3′	5′-TTCTCCGTGGCAATGCCCGTCT-3′	[25]
*Giardia duodenalis* assemblage A	*bp*	5′-CCTCAAGAGCCTGAACGATCTC-3′	5′-AGCTGGTCGTACATCTTCTTCCTT-3′	5‘-TGGC+A+ATGCC+CG+TCT-3‘	[28]
*Giardia duodenalis* assemblage A	*tpi*	5′-CATTGCCCCTTCCGCC-3′	5′-CTGCGCTGCTATCCTCAACTG-3′	5′-CCATTGCGGCAAACA-3′	[29]
*Giardia duodenalis* assemblage B	*bp*	5′-CCTCAAGAGCCTGAACGACCTC-3′	5′-AGCTGGTCATACATCTTCTTCCTC-3′	5′-TTCTCCGTGGCGATGCCTGTCT-3′	[25]
*Giardia duodenalis* assemblage B	*bp*	5′-CCTCAAGAGCCTGAACGACCTC-3′	5′-AGCTGGTCATACATCTTCTTCCTC-3′	5′-TGGCG+ATGC+C+T+GTCT-3′	[28]
*Giardia duodenalis* assemblage B	*tpi*	5′-GATGAACGCAAGGCCAATAA-3′	5′-TCTTTGATTCTCCAATCTCCTTCTT-3′	5′-AATATTGCTCAGCTCGAG-3′	[29]
Phocid herpes virus	*gB*	5′-GGGCGAATCACAGATTGAATC-3′	5′-GCGGTTCCAAACGTACCAA-3′	5′-TTTTTATGTGTCCGCCACCATCTGGATC-3′	[48]

+ = following base is LCA (locked nucleic acid).

**Table A2:** Details of the run conditions of the compared PCR assays. All assays were run with HotStar Taq Mastermix (Qiagen, Hilden, Germany).

	Run Conditions for All Three *G. duodenalis*-Specific Screening Assays	Run Conditions for the Assemblage-Specific Assays Targeting the *bg* Gene without Locked Nucleic Acids	Run Conditions for the Assemblage-Specific Assays Targeting the *bg* Gene with Locked Nucleic Acids	Run Conditions for the Assemblage-Specific Assays Targeting the *tri* Gene
Reaction chemistry				
Reaction volume (µL)	20	20	20	20
Forward primer concentration (pmol/µL)	12.5 (18S rRNA gene), 20 (*gdh* gene), 30 (*bp* gene)	30 (both assemblages)	30 (both assemblages)	30 (both assemblages)
Reverse primer concentration (pmol/µL)	12.5 (18S rRNA gene), 20 (*gdh* gene), 30 (*bp* gene)	30 (both assemblages)	30 (both assemblages)	90 (both assemblages)
Probe concentration (pmol/µL)	1 (18S rRNA gene), 20 (*gdh* gene), 0.625 (*bp* gene)	20 (both assemblages)	20 (both assemblages)	10 (both assemblages)
Final Mg^2+^ concentration (nM)	3	4	3	3
Bovine serum albumin (ng/µL)	100	100	100	-
Run conditions				
Initial denaturation	15 min 95 °C	15 min 95 °C	15 min 95 °C	15 min 95 °C
Cycle numbers	40	40	50	50
Denaturation	15 sec 95 °C	15 sec 95 °C	10 sec 95 °C	15 sec 95 °C
Annealing	Combined annealing/amplification: 60 sec 60 °C	Combined annealing/amplification: 60 sec 60 °C	8 sec 58 °C	Combined annealing/amplification: 60 sec 60 °C
Amplification			3 sec 72 °C	
Hold	10 sec 40 °C	10 sec 40 °C	10 sec 40 °C	10 sec 40 °C

sec = second, min = minute, °C = degree centigrade.

**Table A3:** Sequence insert of the positive control plasmid.

Positive Control Insert Based on *G. duodenalis* Sequences According to the NCBI GenBank Accession Numbers M54878, KJ499992, M36728, AY258616, L02120, and L02116.
5′-GAATTCGGACGCGGCGGACGGCTCAGGACAACGGTTGCACCCCCCGCGGCGGTCCCTGCTAGCCGGACACCGCTGGCAACCCGGCGCCAGAATTCTCGAGCAGATCCTGAAGAACTCCCTCACCACGCTCCCGATGGGCGGCGGCAAGGGCGGCTCCGACTTTGACCCAAAGGGCAAGTCCGACAACGAGGTCATGCGCTTCTGCCAGTCCTTCGAATTCCGTTCGAGGACATCCGCGAGGAGGTCAAGAAGTCCGCCGACAACATGTACCTAACGATCAAGGAGGAGATCGACACCATGGCTGCAAACTTCCGCGAATTCGGAAGGAGGCCCTCAAGAGCCTGAACGATCTCGAGACGGGCATTGCCACGGAGAACGCAGAAAGGAAGAAGATGTACGACCAGCTCAACGAGAAGGAATTCGGAAGGAGGCCCTCAAGAGCCTGAACGACCTCGAGACAGGCATCGCCACGGAGAACGCCGAGAGGAAGAAGATGTATGACCAGCTCAACGAGAAAGAATTCTGGACGTCGTCATTGCCCCTTCCGCCGTACACCTGTCAACAGCCATTGCGGCAAACACGTCAAAACAGTTGAGGATAGCAGCGCAGAATGTGTACCGAATTCAGAGACCCTGGATGAACGCAAGGCCAATAACACTATGGAGGTGAATATTGCTCAGCTCGAGGCTCTTAAGAAGGAGATTGGAGAATCAAAGAAGTTATGGGAGAATTCAATTTTGGGCGAATCACAGATTGAATCTGATGATACAGCAACATTTTTTATGTGTCCGCCACCATCTGGATCAACGTTGGTACGTTTGGAACCGCCTCGGGCGAATTC-3′

**Table A4:** Cross-table detailing mismatches between the real-time screening PCR assays targeting *G. duodenalis*. Green = matching results. Red = mismatching results. Black = not filled in to avoid repetition.

		18S rRNA gene		*gdh*		*bg*	
		Negative	Positive	Negative	Positive	Negative	Positive
18S rRNA gene	negative	809					
	positive		63				
*gdh*	negative	747	52	799			
	positive	62	11		73		
*bg*	negative	809	43	780	72	852	
	positive	0	20	19	1		20

**Table A5:** Cross-table detailing mismatches between the real-time differentiation PCR assays targeting the *G. duodenalis* assemblage A. Green = matching results. Red = mismatching results. Black = not filled in to avoid repetition.

		*bg* of Assemblage A without LNA		*bg* of Assemblage A with LNA		*tri* of Assemblage A	
		Negative	Positive	Negative	Positive	Negative	Positive
*bg* of assemblage A without LNA	negative	45					
	positive		8				
*bg* of assemblage A with LNA	negative	8	0	44			
	positive	1	44		9		
*tri* of assemblage A	negative	44	0	43	1	44	
	positive	1	8	1	8		9

**Table A6:** Cross-table detailing mismatches between the real-time differentiation PCR assays targeting the *G. duodenalis* assemblage B. Green = matching results. Red = mismatching results. Black = not filled in to avoid repetition.

		*bg* of Assemblage B without LNA		*bg* of Assemblage B with LNA		*tri* of Assemblage B	
		Negative	Positive	Negative	Positive	Negative	Positive
*bg* of assemblage B without LNA	negative	25					
	positive		28				
*bg* of assemblage B with LNA	negative	21	1	22			
	positive	4	27		31		
*tri* of assemblage B	negative	25	5	21	9	30	
	positive	0	23	1	22		23
